# PD1 BLOCKADE IMPROVES SURVIVAL AND CD8+ CYTOTOXIC CAPACITY, WITHOUT INCREASING INFLAMMATION, DURING NORMAL MICROBIAL EXPERIENCE IN OLD MICE

**DOI:** 10.1093/geroni/igae098.1286

**Published:** 2024-12-31

**Authors:** Christina Camell

**Affiliations:** Institute on the Biology of Aging and Metabolism, University of Minnesota, Province, Minnesota, United States

## Abstract

The elderly and those with chronic diseases have a 2 to 10-fold increased risk of hospitalization and mortality following infection as compared with healthy adults. A significant contributor to disease severity is the aging of the immune system, including an expansion of exhausted T cells. These cells simultaneously promote excessive levels of inflammation and display impaired T cell activation and function. How exhausted T cells from aged individuals respond to infection and whether acute restoration of T cell activation further exacerbates the inflammatory environment or generates functional T cells is unclear. Previously, we used a mouse model of normal microbial experience (NME), where old specific pathogen-free (SPF) mice show 100% mortality following exposure to multiple microbes ordinarily found in pet store mice. In the present study, we show that when exposed to NME, old SPF mice have increased expression of multiple inflammatory pathways and exhibit elevated frequencies of a heterogenous exhausted CD8+ T cell pool that expresses differing combinations of the inhibitory receptor programmed cell death protein 1 (PD1), TOX, and CXCR5. Pre-treatment or intervention with an anti-PD1 monoclonal blocking antibody during the exposure of old SPF mice to NME significantly improves both antibody production and survival, without altering the acute inflammatory response. Anti-PD1 checkpoint blockade-mediated survival is dependent on CD8+, but not CD4+ T cells. Correspondingly, CD8+ PD1+ T cells from old mice given anti-PD1 monoclonal antibody have increased granzyme B production. These data reveal a new approach for reducing vulnerability to infections in the elderly by targeting CD8+ T cell exhaustion through PD1 checkpoint blockade immunotherapy.

